# A longitudinal mixed methods study on changes in body weight, body composition, and lifestyle in breast cancer patients during chemotherapy and in a comparison group of women without cancer: study protocol

**DOI:** 10.1186/s12885-018-5207-7

**Published:** 2019-01-05

**Authors:** J. Th. C. M. de Kruif, M. Visser, M. M. G. A. van den Berg, M. J. M. Derks, M. R. de Boer, H. W. M. van Laarhoven, J. H. M. de Vries, Y. C. de Vries, E. Kampman, R. W. Winkels, M. J. Westerman

**Affiliations:** 1 Department of Health Sciences, Faculty of Science, the Amsterdam Public Health Institute, Vrije Universiteit Amsterdam, Amsterdam, The Netherlands; 2 Division of Human Nutrition and Health, Wageningen University, Wageningen, the Netherlands; 3 Department of Medical Oncology, Academic Medical Center, Cancer Center Amsterdam, Amsterdam, the Netherlands; 4 Department of Public Health Sciences, Penn State College of Medicine, Hershey, PA USA

**Keywords:** Breast cancer, Mixed methods, Perceptions, Body weight, Body composition, Dietary intake, Physical activity, Quality of life

## Abstract

**Background:**

More than 60% of women diagnosed with early stage breast cancer receive (neo)adjuvant chemotherapy. Breast cancer patients receiving chemotherapy often experience symptoms such as nausea, vomiting and loss of appetite that potentially affect body weight and body composition. Changes in body weight and body composition may detrimentally affect their quality of life, and could potentially increase the risk of disease recurrence, cardiovascular disease and diabetes. To date, from existing single method (quantitative or qualitative) studies is not clear whether changes in body weight and body composition in breast cancer patients are treatment related because previous studies have not included a control group of women without breast cancer.

**Methods:**

We therefore developed the COBRA-study (Change Of Body composition in BReast cancer: All-in Assessment-study) to assess changes in body weight, body composition and related lifestyle factors such as changes in physical activity, dietary intake and other behaviours. Important and unique features of the COBRA-study is that it used I) a “Mixed Methods Design”, in order to quantitatively assess changes in body weight, body composition and lifestyle factors and, to qualitatively assess how perceptions of women may have influenced these measured changes pre-, during and post-chemotherapy, and II) a control group of non-cancer women for comparison. Descriptive statistics on individual quantitative data were combined with results from a thematic analysis on the interviews- and focus group data to understand patients’ experiences before, during and after chemotherapy.

**Discussion:**

The findings of our mixed methods study, on chemotherapy treated cancer patients and a comparison group, can enable healthcare researchers and professionals to develop tailored intervention schemes to help breast cancer patients prevent or handle the physical and mental changes they experience as a result of their chemotherapy. This will ultimately improve their quality of life and could potentially reduce their risk for other co-morbidity health issues such as cardiovascular disease and diabetes.

## Background

Breast cancer is the most common cancer in women worldwide and makes up to 25% of all female cancers [1]. Due to early detection through screening programs and therapeutic improvements, the five-year survival rate in the Netherlands has increased from 78 to 88% during the last two decades [2–5]. This implies that the number of breast cancer survivors will steadily increase in the future. The impact of chemotherapy on general health, therefore becomes more important to the health care system.

For breast cancer patients, the side-effects of chemotherapy can be both short- and long term. Regularly reported short-term side-effects include; nausea, vomiting, hair loss, loss of energy and fatigue [6], taste and smell alterations [7–11] psychological distress [12–14] and even chemotherapy-related-hospitalizations [15]. Long-term side-effects of chemotherapy include; psychological distress and physical effects such as fatigue and loss of energy, weight gain [16–18], and unfavourable changes in body composition (increase in fat mass and loss of muscle mass) [17, 19–21] and loss of muscle strength [22, 23]. Weight gain and changes in body composition may have a profound negative influence on quality of life and self-esteem in breast cancer survivors and may also increase the risk of several co-morbidities, such as cardio-vascular disease [24, 25], diabetes [26] and breast cancer recurrence [27–30]. Gaining a better understanding of the processes that underlie these short- and long-term side effects is critical to enable the development of tailored intervention schemes.

To date, studies on these short- and long-term side effects have had relatively insular focuses. Several studies reported that chemotherapy is associated with weight gain. The earlier studies report large weight changes [31, 32], while the more recent reports suggest less weight gain [17–19]. In our meta-analysis [33] we found an overall body weight increase during chemotherapy of 2,7 kg (95% CI 2.0, 7.5) with a high degree of variation: some women gain more than 10 kg while others lose weight. Changes in body weight, body composition and muscle strength among women with breast cancer undergoing chemotherapy were possible influenced by lifestyle factors, such as physical activity and dietary intake and the perception of women on these factors.

Patients are often forced to adapt their daily activities during treatment [34]. Several studies suggest that reductions in physical activity during chemotherapy may contribute to weight gain [9, 21, 35], lower quality of life [36] and increase the risk of disease recurrence [37–40]. Women may be influenced in their decision to engage in physical activity in a negative way [41] e.g. through pressure from friends and family to rest and not to be active [41], or due to lack of time because of taking care of children [42], lack of motivation [43], the side effects of chemotherapy [44], the need to conserve energy, fear, possible injury [45], and difficulty to stay focused during physical activity because of “chemo brain” [46]. When breast cancer patients tried to be more physically active during therapy however, most of them experienced increased wellbeing and restored energy levels during physical activity [47].

Reports on the influence of changes in dietary intake during chemotherapy in cancer patients and how these changes influence their body weight and body composition, both short and long term, vary widely. The literature findings describe that: they showed no changes [7, 21, 48], increases [49]), or decreases [32, 50] in energy intake during chemotherapy. These variations could be due to the different designs of the studies and time points of measurements.

In a recent study by our research group [51] a 10% lower energy intake through dietary changes (including an absolute lower intake of protein, fat and alcohol) for women with breast cancer, was observed during chemotherapy treatment (*n* = 117) based on 24 h recalls [51]. Furthermore, these breast cancer patients scored significantly lower on their self-reported taste, smell, appetite and hunger questionnaires. These results could potentially be due to chemotherapy induced symptoms such as, a dry mouth, lack of energy, nausea and difficulties with chewing [51]. In qualitative studies, patients with breast cancer stated during interviews that they also experienced a decreased enjoyment of food and a change in the role of food: eating for the sake of eating and use of comfort food as a reward, because of these changes in taste and smell [52].

Symptoms of psychological distress are twice as high among breast cancer patients in comparison with the general female population. The impact of breast cancer may have a long-term effect, extending for years after diagnosis [53, 54]. Dealing with the diagnosis ‘breast cancer’ could have a profound influence on their perceptions on changes in body composition and weight related lifestyle factors. Despite available information and guidelines, studies suggest that women hardly experience support in their struggle to deal with the diagnosis and treatment [55–58]. Many women experience psychological stress and impaired quality of life as a result of a breast cancer diagnosis and treatment [6, 13, 59, 60]. Studies suggest that women’s overall health and their altered bodies are constant reminders of their illness and its treatment [9, 16, 52, 55–58, 61–64]. Women reported that they feel frustrated not being able to control their weight [63] and their dietary intake [52]. Although for most women weight management during treatment has lower priority [65], they have to cope psychologically with their diagnosis and the effects of the treatment.

From the current literature, it is clear that for breast cancer patients, the side-effects of chemotherapy can be both short- and long term. Our understanding of the processes that underlie these short- and long-term side effects is however, still incomplete. In what way the patients´ perceptions on lifestyle factors, such as changes in physical activity and dietary intake, influence changes in the body weight and body composition of breast cancer patients is inconsistent and unclear. Furthermore, to date, the majority of these previous studies [7, 17, 19, 21, 63] only assessed changes in patients undergoing chemotherapy during treatment and did not include a comparison group of women without breast cancer.

We developed the COBRA study to objectively assess changes in body weight and body composition and related lifestyle factors and how perceptions of patients influence these factors.). A unique feature of the COBRA study is that it was designed as a longitudinal “Mixed Methods Study” combining both quantitative and qualitative research methods and data. The publications of the COBRA study thus far focused on the quantitative design and quantitative findings of the study (33). The main aim of this manuscript is to describe the methodological design of the qualitative part of the COBRA-study and how the quantitative and qualitative data can be combined in a mixed-methods approach. This enables us to not only quantitatively assess changes in body weight and body composition and related lifestyle factors, but also to qualitative assess how perceptions of breast cancer patients influence these factors not only during chemotherapy, but also pre-, and post-chemotherapy. Furthermore, a group of women without breast cancer are also assessed as a comparison group, the majority of studies so far did not include a comparison group, to evaluate the significance of the breast cancer patient’s results.

## Design and methods

To prepare the study protocol, we conducted a qualitative pilot study among 20 breast cancer patients who had already completed their chemotherapy, in order to gain insight into their experiences with diagnosis and treatment. We learned from these patients that their experience of having cancer influenced their attitude towards quality of life, physical activity and nutrition, beyond the direct effects of chemotherapy such as nausea, vomiting, hair loss and loss of energy. Results from the pilot study showed that all breast cancer patients expressed an urgent need for information concerning nutrition and physical activity during chemotherapy. The pilot study also suggested different results based on age and BMI group; older women and women with BMI > 25 kg/m2 had a less urgent need for this information and they were physically less active when compared to younger women or women with a BMI < 25 kg/m2. We also found that women were sometimes able to come up with solutions to meet their own needs when they were confronted with changes in dietary intake, physical activity and quality of life during chemotherapy. These pilot study results confirmed the relevance of performing an in-depth study because patients expressed an urgent need for information about nutrition and physical activity.

### Mixed-method design

We designed a longitudinal observational, mixed-method approach, to understand patients’ experiences before, during and after chemotherapy, using repeated measurements and interviews as well as focus group meetings (Table 1). The purpose of pairing qualitative and quantitative components [66, 67] within this study is to provide a better understanding of the changes in body weight and body composition. Qualitative measurements of the perception of women on physical activity and dietary intake, as well as factors related to coping with diagnosis and treatment, can help to explain and interpret quantitative measurements of the factors influencing changes in body weight and body composition. A mixed method study is therefore, a good approach to obtain in-depth information and knowledge of the problem (i.e. changes in body weight and body composition) and also provides comprehensive datasets [68]. In addition, this approach assists in increasing the reliability and credibility of the findings through the combination of quantitative and qualitative results, the methodological triangulation [69].

**Table 1:** An overview of all measurements and timing of the COBRA mixed method study

	Time	*Quantitative measurements* General questionnaire Body composition, body weight, muscle strength Energy intake (FFQ) and taste and smell Physical activity^2^ Quality of life, fatigue, depressive symptoms	*Quantitative measurements* Energy intake taste and smell, 24 h recall^1^,	*Qualitative measurements* Interviews *	*Qualitative measurements* focus group meetings**
Women with early breast cancer *n* = 200	T1 pre CT	x		X (*n* = 25)	
	T2 mid-way CT		x	x	
	T3 post CT	x		x	
	T4 6 months post CT	x		x	
	T5 1 yr. post CT				x
Women without breast cancer Comparison group n = 200	T1 inclusion	x		X (*n* = 15)	
	T2 after 3 months		x		
	T3 after 6 months	x			
	T4 after 12 months	x		x	

^1^at two randomly chosen days during CT

^2^accelerometer recordings over a 7 days period

*in a sample of participants (see text for a further description of details)

**focus groups with interviewed and non-interviewed participants
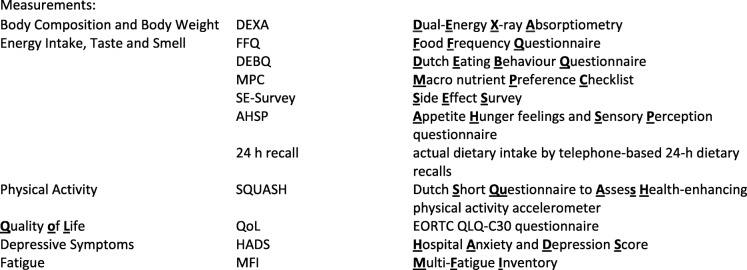

For breast cancer patients, data collection took place four times during this study; T1: pre-chemotherapy, T2: midway chemotherapy, T3: post-chemotherapy (1–3 weeks after last chemotherapy cycle), and T4: half a year post chemotherapy. For the non-breast cancer (comparison) group, data collection took place at: T1: at inclusion, T2 after 3 months, T3 after 6 months, T4 after 1 year. For an overview see Table 1. Approval for the COBRA-study was obtained from the Medical Ethics Committee of the Wageningen University, The Netherlands (ABR NL40666.081.12) and the Scientific Advisory Committee VUMC/VU.

### Participants and recruitment

Two hundred patients with breast cancer, indicated for (neo)adjuvant chemotherapy were recruited from 11 hospitals in the Netherlands. Inclusion criteria were 1) women > 18 years old, 2) newly diagnosed, non-advanced (I-IIIA) operable breast cancer scheduled for initiating 2nd or 3rd generation adjuvant or neo-adjuvant chemotherapy, and 3) able to communicate in Dutch. An exclusion criterion was pregnancy or intentions to become pregnant within the study period. The comparison group of women without any history of cancer was recruited via the women with breast cancer, who were asked to distribute information about the study to female friends, acquaintances and colleagues of the same age or 2 years younger or older. Women without cancer contacted the researchers if they were interested in participating in the study. We recruited 200 women for this comparison group. All respondents signed a written informed consent.

For the mixed method part of the COBRA-study, a subgroup of *N* = 25 breast cancer patients was selected for the qualitative part of the study (Table 1). Purposive sampling was applied to reach as wide a range of perspectives as possible, and to capture the broadest set of information and experiences. Based on previous literature and the results from our pilot study, we used the following criteria for this sampling: variation in age (25-64 yr), pre- or postmenopausal status (pre *n* = 10, peri *n* = 3, post *n* = 12), Body Mass Index (BMI) > 25 kg/m^2^ (*n* = 11) or < 25 (*n* = 14), and stage I to IIIa breast cancer. With the exception of the last criteria, the comparison group of women without breast cancer (*n* = 15) were selected using the same criteria.

### Data collection used for the mixed method study

#### Quantitative data collection

Body composition, body weight and dietary intake were assessed using: 1) a total body Dual-Energy X-ray absorptiometry (DEXA) scan, 2) a Food Frequency Questionnaire (FFQ) [70] on energy intake, 3) two telephone-based 24-h dietary recalls during chemotherapy for actual dietary intake because of the expected high day to day variation during chemotherapy treatment and, 4) the Appetite Hunger Feelings and Sensory Perception Questionnaire (AHSP) [71] on appetite, hunger, taste and smell which was assessed with additional questions about the severity of 13 key symptoms that often occur during chemotherapy. Physical activity level was assessed by the Dutch Short Questionnaire to Assess Health-enhancing physical activity (SQUASH) [72] and by an accelerometer which the women wore for 7 days. Quality of life was assessed by the EORTC C-30 questionnaire [73], depression and anxiety by the Hospital Anxiety and Depression Score (HADS) [74], and fatigue by the multi-Fatigue Inventory (MFI) [75]. See Table 1 for a description and the timing of different measurements.

#### Qualitative data collection

##### Interviews

The timing of the interviews is shown in Table 1. Semi-structured interviews were held, guided by a topic list based on a literature review and our pilot study. Potential changes in aspects of dietary intake, physical activity and quality of life from the perspective of the participants were questioned. Patients were asked to elaborate about these topics and to mention all issues relevant from their own perspective. Additional questions were asked to uncover beliefs, values, and motivations that underlie individual health behaviours such as response to diagnosis, physical and mental health, and influences from the social environment during and after chemotherapy. Each of the four interviews at T1, T2, T3 and T4 with every patient, builds on the previous one. Each interview explicitly asks the women how their experiences change over time. Interviews take place at patients’ homes or elsewhere, based on the preferences of the patients.

All interviews are audiotaped and transcribed verbatim. Patients are asked to give feedback on a written summary of the interview to foster validity (member checks). The interviews with the non-breast cancer women in the comparison group, provides us with information to obtain a better understanding of the perception and experiences of the patients during treatment.

#### Focus groups

Focus group sessions were conducted after the interviews to validate, enrich and further explore the data gathered during the interviews of the women with breast cancer (Table 1). In these sessions, we also explored possible strategies the women use to curb identified changes in dietary intake, physical activity, body weight and quality of life.

Since the study has an emergent design, the qualitative study design evolves over time, and, the themes to be discussed in the focus group sessions emerge from the results of the previous personal interviews. For the focus group sessions interviewed patients and non-interviewed patients are invited and, eight to ten respondents participate in the assigned focus groups. The sessions are moderated by a qualified researcher and observed by a second member of the research team. The focus group sessions are recorded on audiotape. The final number of focus group sessions depends on the validation and enrichment of the data.

### Data analysis

#### Qualitative data

Analysis of the interview data starts during data collection. All transcripts of the interviews are analyzed using a thematic content analysis with comparisons within and across the interviewed respondents [76]. The qualitative data analysis software MAXQDA (VERBI software, Marburg, Germany) is used to manage the data [77]. Transcripts are subsequently disentangled and divided into fragments and open-coded. Codes are categorized by subthemes and main themes. Relationships between the subthemes are explored, to eventually cover the subthemes under the overall themes. The codes, subthemes and themes are discussed within the research team until consensus is reached on all the themes. Codes and (sub)themes are structured in a code tree. The constant comparison method [76] is used in order to understand the differences, as well as similarities, between and within women. The main results are discussed within the research team to enhance the robustness of the findings. The themes recognized are used to find answers for the aim of the study, and to describe patterns and mechanisms within the whole dataset to provide a broader overview of the findings.

The data gathered during the individual interviews are validated and enriched in the focus group sessions. Combining these two methods (interviews and focus groups) enabled us to check for inconsistencies and continuities between what was said in individual interviews and what emerged from interactive group discussions.

#### Combined data

Mixed methods is an approach which draws upon the strengths and perspectives of each method: the existence of the natural physical world, quantitative, as well as the reality and influence of human experience, qualitative method [78]. The collection and analysis of both data sets is carried out separately and the findings are not compared or consolidated until the interpretation stage, and finally sequential data analysis. The data are analyzed in a particular sequence with the use of, or findings from, the other method [79]. Quantitative results obtained from the measurements and questionnaires (Table 1) are combined with the qualitative results obtained from the individual interviews and focus group sessions. Together, these data sets can provide a more complete and comprehensive evaluation of the changes in body weight and body composition [80]. Findings generated by the different data collection methods could elucidate aspects of the changes in body weight and body composition allowing us to explore the outcome from the analysis, whether that be convergent, where qualitative and quantitative findings lead to the same conclusion; complementary, where qualitative and quantitative results can be used to supplement each other or; divergent, where the combination of qualitative and quantitative results provides different (and at times contradictory) findings [69, 81].

In this study the quantitative part describes how the body changes during chemotherapy and the period thereafter (biomedical changes). The qualitative part focuses on how women experience potential changes in their body and what role eating and exercise behaviour plays (lifestyle changes). The combination of these two parts (quantitative and qualitative) makes it possible to explain and interpret these body and lifestyle (dietary and physical activity) changes in order to better understand changes in body weight and body composition [80]. Descriptive results of the quantitative measurements such as body weight, body composition, muscle strength, quality of life, smell and taste, and depression and anxiety on an individual level are linked to the results of the interviews and focus groups sessions, in other words, are linked to the women’s perceptions on these issues, as identified by the different themes in the thematic analysis approach. As a result, certain potential changes in body weight and body composition during chemotherapy can be better understood with the help of the perception of women on physical activity, dietary intake and their subsequent lifestyle behaviour.

## Discussion

In this paper we describe the methodological design of the qualitative part of the COBRA-study and how the quantitative and qualitative data can be combined in a mixed-methods approach. To our knowledge, this study is the first longitudinal study in women with breast cancer that combines both qualitative and quantitative methodologies with measurements taken before, during and after chemotherapy. Furthermore, it is the first study to have a control group of non-cancer women for comparison.

This mixed methods study focuses specifically on the quantitative and qualitative changes in body weight and body composition in patients with breast cancer during chemotherapy compared to women without breast cancer. It explores the perceptions of women with and without breast cancer and how they deal with quantitatively measured, changes in body weight, taste and smell, dietary intake, physical activity and quality of life.

Due to the longitudinal nature of the study, the measurements and the perception and experiences of breast cancer patients at various time points; pre-, during and post chemotherapy treatment; can be better understood. Specific time points at which additional support for women is required can be evaluated and defined. The collection of both qualitative and quantitative data facilitates a more complete insight and a better understanding of the changes in body weight, body composition and muscle strength.

The findings of this study will help researchers, health care professionals and the breast cancer patients themselves to understand the struggles women with breast cancer undergoing chemotherapy have, and their needs during their treatment. This information will enable health care professionals to develop practicable, feasible and tailored interventions that could help breast cancer patients to handle or prevent treatment/weight related lifestyle changes and ultimately improve their quality of life and future health.

## Abbreviations

COBRA-study: Change Of Body composition in BReast cancer: All-in Assessment-study

## References

[1] World Cancer Research Fund: Data on specific cancers https://www.wcrf.org/int/cancer-facts-figures/data-specific-cancers/breast-cancer-statistics. Accessed 20 May 2018.

[2] Nederlandse Kankerregistratie. https://www.cijfersoverkanker.nl. Accessed May 4, 2018.

[3] Sukel MPP, van de Poll-Franse LV, Nieuwenhuijzen GAP, Vreugdenhil G, Herings RMC, Coebergh JWW, Voogd AC (2008). Substantial increase in the use of adjuvant systemic treatment for early stage breast cancer reflects changes in guidelines in the period 1990-2006 in the southeastern Netherlands. Eur J Cancer.

[4] Berrino F, De Angelis R, Sant M, Rosso S, Lasota MB, Coebergh JW, Santaquilani M (2007). The EUROCARE working group. Survival for eight major cancers and all cancers combined for European adults diagnosed in 1995-99: results of the EUROCARE-4 study. The Lancet oncology.

[5] Early Breast Cancer Trialists’ Collaborative Group (EBCTCG) (2005). Effects of chemotherapy and hormonal therapy for early breast cancer on recurrence and 15-year survival: an overview of the randomised trials. Lancet.

[6] Bower JE, Ganz PA, Desmond KA (2000). Fatigue in breast cancer survivors: occurrence, correlates and impact on quality of life. J Clin Oncol.

[7] Harvie MN, Campbell IT, Baildam A, Howell A. Energy balance in early breast cancer patients receiving adjuvant chemotherapy. Breast Cancer Res Treat. 2004;(3):201–10. 10.1023/B:BREA.0000014037.48744.fa 14758090

[8] Steinbach S, Hummel T, Bohner C, Berktold S, Hundt W, Kriner M, Heinrich P, Sommer H, Hanusch C, Prechtl A, Schmidt B, Bauerfeind I, Seck K, Jacobs VR, Schmalfeldt B, Harbecket N (2009). Qualitative and quantitative assessment of taste and smell changes in patients undergoing chemotherapy for breast cancer or gynecologic malignancies. J Clin Oncol.

[9] Boltong A, Keast R, Aranda S (2012). Experiences and consequences of altered taste, flavour, and food hedonics during chemotherapy treatment. Support Care Cancer.

[10] Gamper E, Zabernigg A, Wintner LM, Giesinger JM, Oberguggenberger A, Kemmler G, Sperner-Unterweger B, Holzner B. Coming to your senses: detecting taste and smell alterations in chemotherapy patients. A systematic review. J Pain Symptom Manag. 2012;(6):880–94. 10.1016/j.jpainsymman.2011.11.011 22921177

[11] Brisbois TD, de KIH, Watanabe SH, Baracos VE, Wismer WV (2011). Characterization of chemosensory alterations in advanced cancer reveals specific chemosensory phenotypes impacting dietary intake and quality of life. J Pain Symptom Manag.

[12] Burgess C, Cornelius V, Love S, Graham J, Richards M, Ramirez A (2005). Depression and anxiety in women with early breast cancer: five-year observational cohort data. BMJ.

[13] Helms RL, O'Hea EL, Corso M (2008). Body image issues in women with breast cancer. Psychol Health Med.

[14] Kornblith AB, Ligibel J. Psychosocial and sexual functioning of survivors of breast Cancer. Semin Oncol. 2003;(6):799–813. 10.1053/j.seminoncol.2003.08.025 14663780

[15] Barcenas CH, Niu J, Zhang N, Zhang Y, Buchholz TA, Elting LS, Hortobagyi GN, Smith BD, Giordano SH (2014). Risk of hospitalization according to chemotherapy regimen in early-stage breast Cancer. J Clin Oncol.

[16] McInnes JA, Knobf MT (2001). Weight gain and quality of life in women treated with adjuvant chemotherapy for early-stage breast cancer. Oncol Nurs Forum.

[17] Freedman RJ, Aziz N, Albanes D, Hartman T, Danforth D, Hill S, Sebring N, Reynolds JC, Yanovski JA. Weight and body composition changes during and after adjuvant chemotherapy in women with breast Cancer. J Clin Endocrinol Metab. 2004;(5):2248–53. 10.1210/jc.2003-031874 15126549

[18] Makari-Judson G, Judson CH, Mertens WC. Longitudinal patterns of weight gain after breast cancer diagnosis: observations beyond the first year. Breast Journal. 2007;(3):258–65. 10.1111/j.1524-4741.2007.00419.x 17461900

[19] Campbell KL, Lane K, Martin AD, Gelmon KA, McKenzie DC (2007). Resting energy expenditure and body mass changes in women during adjuvant chemotherapy for breast cancer. Cancer Nurs.

[20] Ingram C, Brown JK. Patterns of weight and body composition change in premenopausal women with early stage breast cancer: has weight gain been overestimated? Cancer Nurs. 2004;(6):483–90. 10.1097/00002820-200411000-00008 15632788

[21] Demark-Wahnefried W, Peterson BL, Winer EP, Marks L, Aziz N, Marcom PK, Blackwell K, Rimer BK. Changes in weight, body composition, and factors influencing energy balance among premenopausal breast cancer patients receiving adjuvant chemotherapy. J Clin Oncol. 2001;(9):2381–9. 10.1200/JCO.2001.19.9.2381 11331316

[22] Knols RH, Stappaerts KH, Fransen J, Uebelhart D, Aufdemkampe G (2002). Isometric strength measurement for muscle weakness in cancer patients: reproducibility of isometric muscle strength measurements with a hand-held pull-gauge dynamometer in cancer patients. Support Care Cancer.

[23] Visovsky C (2006). Muscle strength, body composition, and physical activity in women receiving chemotherapy for breast cancer. Integrative Cancer Therapies.

[24] Willett WC, Manson JE, Stampfer MJ, Colditz GA, Rosner B, Speizer FE, Hennekens CH (1995). Weight, weight change, and coronary heart disease in women. Risk within the 'normal' weight range Journal of the American Medical Association.

[25] Nichols HB, Trentham-Dietz A, Egan KM, Titus-Ernstoff L, Holmes MD, Bersch AJ, Hlick CN, Hampton JM, Stampfer MJ, Willett WC, Newcomb PA. Body mass index before and after breast cancer diagnosis: associations with allcause, breast cancer and cardiovascular disease mortality. Cancer Epidemiological Biomarkers and Prevention. 2009;(5):1403–9. 10.1158/1055-9965.EPI-08-1094 PMC2715918 19366908

[26] Erickson KD, Patterson RE, Natarajan L, Lindsay SP, Heath D, Caan BJ (2012). Weight change and risk of incident diabetes after breast cancer. Cancer Res.

[27] Kroenke CH, Chen WY, Rosner B, Holmes MD. Weight, weight gain, and survival after breast cancer diagnosis. J Clin Oncol. 2005;(7):1370–8. 10.1200/JCO.2005.01.079 15684320

[28] Thivat E, Thérondel S, Lapirot O, Abrial C, Gimbergues P, Gadéa E, Planchat E, Kwiatkowski F, Mouret-Reynier MA, Chollet P, Durando X (2010). Weight change during chemotherapy changes the prognosis in non-metastatic breast cancer for the worse. BMC Cancer.

[29] Playdon MC, Bracken MB, Sanft TB, Ligibel JA, Harrigan M, Irwin MLK (2015). Weight gain after breast Cancer diagnosis and all-cause mortality: systematic review and meta-analysis. JNCI J Natl Cancer Inst.

[30] Azrad M, Demark-Wahnefried W (2014). The association between adiposity and breast cancer recurrence and survival: a review of the recent literature. Curr Nutr Rep.

[31] Demark-Wahnefried W, Winer EP, Rimer BK (1993). Why women gain weight with adjuvant chemotherapy for breast cancer. J Clin Oncol.

[32] Demark-Wahnefried W, Rimer BK, Winer EP (1997). Weight gain in women diagnosed with breast cancer. J Am Diet Assoc.

[33] Berg MMGA van den, Winkels RM, Kruif JThCM de, Laarhoven HWM van, Visser M, Vries JHM de, Vries YC de, Kampman E. Weight change during chemotherapy in breast cancer patients: a meta-analysis. BMC Cancer 2017 12;17(1):259. 10.1186/s12885-017-3242-4 10.1186/s12885-017-3242-4 PMC5389147 28403873

[34] Chlebowski RT, Aiello E, McTiernan A (2002). Weight loss in breast cancer patient management. J Clin Oncol.

[35] Irwin ML, Crumley D, McTiernan A, Bernstein L, Baumgartner R, Gilliland FD, Kriska A, Ballard-Barbash R (2003). Physical activity levels before and after a diagnosis of breast carcinoma: the health, eating, activity, and lifestyle (HEAL) study. Cancer.

[36] Montazeri A. Health-related quality of life in breast cancer patients: a bibliographic review of the literature from 1974 to 2007. J Exp Clin Cancer Res. 2008. 10.1186/1756-9966-27-32. 10.1186/1756-9966-27-32 PMC2543010 18759983

[37] Lahart IM, Metsios GS, Nevill AM, Carmichael AR (2015). Physical activity, risk of death and recurrence in breast cancer survivors: a systematic review and meta-analysis of epidemiological studies. Acta Oncol.

[38] Holmes MD, Chen WY, Feskanich D, Kroenke CH, Colditz GA (2005). Physical activity and survival after breast cancer diagnosis. JAMA.

[39] Irwin ML, McTiernan A, Manson JE, Thomson CA, Sternfeld B, Stefanick ML (2011). Physical activity and survival in postmenopausal women with breast cancer: results from the Women’s health initiative. Cancer Prev Res.

[40] Ibrahim EM, Al-Homaidh A (2011). Physical activity and survival after breast cancer diagnosis: a meta-analysis of published studies. Med Oncol.

[41] Brunet J, Taran S, Burke S, Sabiston CM (2013). A qualitative exploration of barriers and motivators to physical activity participation in women treated for breast cancer. Disabil Rehabil.

[42] Larsson IL, Jonsson C, Olsson AC, Gard G, Johansson K (2008). Women’s experience of physical activity following breast cancer treatment. Scand J Caring Sci.

[43] Loh SY, Chew SL, Lee SY (2011). Physical activity and women with breast cancer: insights from expert patients. Asian Pac J Cancer Prev.

[44] Ingram C, Wessel J, Courneya KS (2010). Women’s perceptions of home-based exercise performed during adjuvant chemotherapy for breast cancer. Eur J Oncol Nurs.

[45] Husebø AM, Karlsen B, Allan H, Søreide JA, Bru E (2015). Factors perceived to influence exercise adherence in women with breast cancer participating in an exercise programme during adjuvant chemotherapy: a focus group study. J Clin Nurs.

[46] Balneaves LG, Van Patten C, Truant TL, Kelly MT, Neil SE, Campbell KL (2014). Breast cancer survivors’ perspectives on a weight loss and physical activity lifestyle intervention. Support Care Cancer.

[47] Backman M, Browall M, Sundberg CJ, Wengström Y (2016). Experiencing health: physical activity during adjuvant chemotherapy treatment for women with breast cancer. Eur J Oncol Nurs.

[48] Del Rio G, Zironi S, Valeriani L, Menozzi R, BondiM BM, Piccinini L, Banzi MC, Federico M (2002). Weight gain in women with breast cancer treated with adjuvant cyclophosphomide, methotrexate and 5-fluorouracil. Analysis of resting energy expenditure and body composition. Breast Cancer Res Treat.

[49] Grindel CG, Cahill CA, Walker M (1989). Food intake of women with breast cancer during their first six month of chemotherapy. Oncol Nurs Forum.

[50] Custódio IDD, Marinho EDC, Gontijo CA, Pereira TSS, Paiva CE, De Maia YCP. Impact of chemotherapy on diet and nutritional status of women with breast cancer: a prospective study. PLoS One. 11(6):e0157113. 10.1371/journal.pone.0157113. 10.1371/journal.pone.0157113 PMC4911080 27310615

[51] Vries YC de, van den Berg MMGA, de Vries JHM, Boesveldt S, de Kruif JTCM, Buist N, Haringhuizen A, Los M, Sommeijer DW, Timmer-Bonte JHN, van Laarhoven HWM, Visser M, Kampman E, Winkels RM. Differences in dietary intake during chemotherapy in breast cancer patients compared to women without cancer. Support Care Cancer. 2017 Aug;25(8):2581–2591. 10.1007/s00520-017-3668-x. Epub 2017 Mar 16. 10.1007/s00520-017-3668-x PMC5486772 28303381

[52] Kwok A, Palermo C, Boltong A (2015). Dietary experiences and support needs of women who gain weight following chemotherapy for breast cancer. Support Care Cancer.

[53] Mertz BG, Bistrup PE, Johansen C, Dalton SO, Deltour I, Kehlet H, Kroman N (2012). Psychological distress among women with newly diagnosed breast cancer. Eur J Oncol Nurs.

[54] Soo H, Sherman KA (2015). Rumination, psychological distress and post-traumatic growth in women diagnosed with breast cancer. Psychooncology.

[55] Vinokur AD, Threatt BA, Vinokur-Kaplan D, Stariano WA (1990). The process of recovery from breast cancer for younger and older patients: changes during the first year. Cancer.

[56] Psychological Aspects of Breast Cancer Study Group (1987). Psychological response to mastectomy: a prospective comparison study. Cancer.

[57] Kaptein AA, Schoones JW, Fischer MJ, Thong MSY, Kroep JR, van der HKJM (2015). Illness perceptions in women with breast cancer. A systematic literature review. Curr Breast Cancer Rep.

[58] Ganz PA, Kwan L, Stanton AL, Bower JE, Belin TR (2011). Physical and psychosocial recovery in the year after primary treatment of breast cancer. J Clin Oncol.

[59] Rock CL, Flatt SW, Newman V, Caan BJ, Haan MN, Stefanick ML, Faerber S, Pierce JP (1999). Factors associated with weight gain in women after diagnosis of breast cancer. J Am Diet Assoc.

[60] DeGeorge D, Gray JJ, Fetting JH, Rolls BJ (1990). Weight gain in patients with breast cancer receiving adjuvant treatment as a function of restraint, disinhibition. and hunger Oncol Nurs Forum.

[61] Kutynec CL, McCargar L, Barr SI, Hislop TG. Energy balance in women with breast cancer during adjuvant treatment. J Am Diet Assoc. 1999;(10):1222–7. 10.1016/s0002-8223(99)00301-6 10524385

[62] Golant M, Altman T, Martin C (2003). Managing cancer side effects to improve quality of life. Cancer Nurs.

[63] Halbert CH, Weathers B, Esteve R, Audrain-McGovern J, Kumanyika S, DeMichele A, Barg F. Experiences with weight change in African-American breast cancer survivors. Breast J. 2008;(2):182–7. 10.1111/j.1524-4741.2007.00551.x 18282235

[64] Avis NE, Crawford S, Manuel J (2005). Quality of life among younger women with breast cancer. J Clin Oncol.

[65] Vance V, Mourtzakis M, McCargar L, Hanning R (2011). Weight gain in breast cancer survivors: prevalence, pattern and health consequences. Obes Rev.

[66] Tashakkori A, Teddlie C. Handbook of mixed methods in Social & Behavioural Research. Thousands oaks: Sage; 2003.

[67] Ostlund U, Kidd L, Wengstrom Y, Rowa-Dewar N (2011). Combining qualitative and quantitative research within mixed method research designs: a methodological review. Int J Nurs Stud.

[68] Ruffin MT, Creswell JW, Jimbo M, Fetters MD (2009). Factors influencing choices for colorectal Cancer screening among previously unscreened African and Caucasian Americans: findings from a triangulation mixed methods investigation. J Community Health.

[69] Erzberger C, Kelle U, Tashakkori A, Teddlie C (2003). Making inferences in mixed methods: the rules of integration. Handbook of mixed methods in Social & Behavioural Research.

[70] Streppel MT, de Vries JH, Meijboom S, Beekman M, de Craen AJ, Slagboom PE, Feskens EJ (2013). Relative validity of the food frequency questionnaire used to assess dietary intake in the Leiden longevity study. Nutr J.

[71] Mathey MFAM, De Jong N, De Groot CPGM, De Graaf C, Van Staveren WA (2001). Assessing appetite in Dutch elderly with the appetite, hunger and sensory perception (AHSP) questionnaire. Journal of Nutrition, Health and Aging.

[72] Wendel-Vos GC, Schuit AJ, Saris WH, Kromhout D (2003). Reproducibility and relative validity of the short questionnaire to assess health-enhancing physical activity. J Clin Epidemiol.

[73] Aaronson N, Ahmedzai S, Bergman B (1993). The European Organization for Research and Treatment of Cancer QLQ-C30: a quality-of-life instrument for use in international clinical trials in oncology. J Natl Cancer Inst.

[74] Annunziata MA, Muzzatti B, Altoe G (2011). Defining hospital anxiety and depression scale (HADS) structure by confirmatory factor analysis: a contribution to validation for oncological settings. Ann Oncol.

[75] Gentile S, Delarozière JC, Favre F, Sambuc R, San Marco JL (2003). Validation of the French multidimensional fatigue inventory (MFI 20). European Journal of Cancer Care.

[76] Braun V, Clarcke V. Successful qualitative research. Sage publications ltd 2013.

[77] MAXQDA, software for qualitative data analysis. (1989–2015). Berlin, Germany: VERBI Software - Consult - Sozialforschung GmbH.

[78] Johnson RB, Onwuegbzie AJ. Mixed methods research: a research paradigm whose time has come. Educ Res. 2004;(7):14–26.

[79] Onwuegbuzie A J, and C Teddlie 2003. A framework for analyzing data in mixed methods research. In handbook of mixed methods in social and behavioral research. In: a Tashakkori and C Teddlie, editors. Thousand Oaks: Sage 2003. p. 351–383.

[80] Johnson R, Onwuegbuzie A, Turner L (2007). Toward a definition of mixed methods research. J Mix Methods Res.

[81] Greene J, Caracelli V, Graham W (1989). Toward a conceptual framework for mixedmethod evaluation designs. Educ Eval Policy Anal.

